# Radionuclide Analysis on Bamboos following the Fukushima Nuclear Accident

**DOI:** 10.1371/journal.pone.0034766

**Published:** 2012-04-04

**Authors:** Takumi Higaki, Shogo Higaki, Masahiro Hirota, Kae Akita, Seiichiro Hasezawa

**Affiliations:** 1 Department of Integrated Biosciences, Graduate School of Frontier Sciences, The University of Tokyo, Kashiwanoha, Kashiwa, Chiba, Japan; 2 Advanced Measurement and Analysis, Japan Science and Technology Agency, Chiyoda-ku, Tokyo, Japan; 3 Radioisotope Center, The University of Tokyo, Yayoi, Bunkyo-ku, Tokyo, Japan; 4 Department of Nuclear Engineering and Management, The University of Tokyo, Yayoi, Bunkyo-ku, Tokyo, Japan; National Taiwan University, Taiwan

## Abstract

In response to contamination from the recent Fukushima nuclear accident, we conducted radionuclide analysis on bamboos sampled from six sites within a 25 to 980 km radius of the Fukushima Daiichi nuclear power plant. Maximum activity concentrations of radiocesium ^134^Cs and ^137^Cs in samples from Fukushima city, 65 km away from the Fukushima Daiichi plant, were in excess of 71 and 79 kBq/kg, dry weight (DW), respectively. In Kashiwa city, 195 km away from the Fukushima Daiichi, the sample concentrations were in excess of 3.4 and 4.3 kBq/kg DW, respectively. In Toyohashi city, 440 km away from the Fukushima Daiichi, the concentrations were below the measurable limits of up to 4.5 Bq/kg DW. In the radiocesium contaminated samples, the radiocesium activity was higher in mature and fallen leaves than in young leaves, branches and culms.

## Introduction

On March 11, 2011, several large earthquakes hit the northeast coast of Honshu, Japan, followed by a massive tsunami along the east coastline of Japan. The tsunami that damaged the Tokyo Electric Power Company (TEPCO) Fukushima Daiichi nuclear power plants caused huge releases of radionuclides into the environment of Japan [1]. However, the accident also had global effects as suggested by the detection of radioxenon (^133^Xe) in Washington, USA [2] and Vancouver, Canada [3], and of radioiodine (^131^I) and radiocesium (^134^Cs, ^137^Cs) in California and Washington, USA [4], Thessaloniki, Greece [5], Bremen, Germany [6], Huelva, Spain [7] and Krasnoyarsk, Russia [8]. The spread and effects of the ^134^Cs and ^137^Cs radionuclides have become of prime interest because of their dose and long half-lives of 2 and 30 years, respectively. Indeed, we are faced by the serious problem of radiocesium attachment to and/or uptake by agricultural plants [9].

Bamboo is a fast-growing renewable biomass that is widely distributed throughout Asia. In Japan, the above-ground biomass of *Phyllostachys pubescens* has been estimated at 116.5 t dry matter ha^−1^ for culms and with a gross annual soil respiration of 52.3 t dry CO_2_ ha^−1^ [10]. The large quantities of bamboo leaves that are often used for bamboo grass tea or as cattle food are indicative of their huge environmental impact. Also, the fallen leaves that are spread in the environment might enter the human food chain via soil decomposer. Here, we report the results of the radionuclide analysis on bamboos following the Fukushima nuclear accident.

## Methods

### Ethics Statement

No specific permits were required for the described field studies: a) no specific permissions were required for these locations/activities; b) location are not privately-owned or protected; c) the field studies did not involve endangered or protected species.

### Plant samples

**Figure 1:**
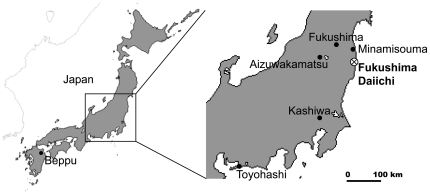
Map of Japan, showing the six locations investigated and the Fukushima nuclear plant.

We sampled leaves and branches of *Phyllostachys nigra* var. henonis Staph (Hachiku) in Minamisouma city, Fukushima Prefecture (25 km from Fukushima Daiichi) on 24th July 2011; *Pleioblastus chino* Makino (Azumanezasa) in Fukushima city, Fukushima Prefecture (65 km from Fukushima Daiichi) on 24th July 2011; *Phyllostachys aurea* Carr. Ex A. Riv. Et C (Hoteichiku) in Aizuwakamatsu city, Fukushima Prefecture (100 km from Fukushima Daiichi) on 24th July 2011; *Pleioblastus simonii* Nakai (Medake) in Kashiwa city, Chiba Prefecture (195 km from Fukushima Daiichi) on 1st, July, 21st July and 24th August 2011; *Pleioblastus simonii* Nakai (Medake) in Toyohashi city, Aichi Prefecture (440 km from Fukushima Daiichi) on 20th August 2011; and *Pseudosasa japonica f. pleioblastoides* Muroi (Menyadake) in Beppu city, Oita Prefecture (980 km from Fukushima Daiichi) on 5th September 2011 (Fig. 1). New leaves described in this report were estimated to have emerged approximately two weeks before collection. The heights of all bamboo at collection were 3 to 5 m. Before radioactive measurements, all samples were dried at 60°C (dry oven, SANYO, MOV-112S) for 24 hours.

### Radioactive measurements

The samples were analyzed by gamma spectrometry, equipped with a high purity germanium detector (Princeton Gamma-Tech, IGC-30180) and a multi-channel analyzer (Canberra, DSA-1000). The detector was shielded with 5 cm of lead to reduce background contributions from the surroundings. For determination of ^134^Cs activity concentrations, gamma-ray energies of 604.70 and 795.85 keV were used. The ^137^Cs activity concentrations were determined from the 661.66 keV peak energies. The samples were measured for a period of 7,200 seconds.

We cite data of the air dose rate 1 m above the ground surface for Minamisouma, Fukushima, and Aizuwakamatsu cities, opened by the Japanese Ministry of Education, Culture, Sports, Science and Technology (MEXT) [11]; the data for Kashiwa city, opened by The University of Tokyo [12]; the data for Toyohashi city, opened by Aichi Prefecture [13]; and the data for Beppu city, opened by Oita Prefecture [14]. All air dose rates were measured using portable survey meters from late June to mid-August, 2011.

## Results

**Figure 2:**
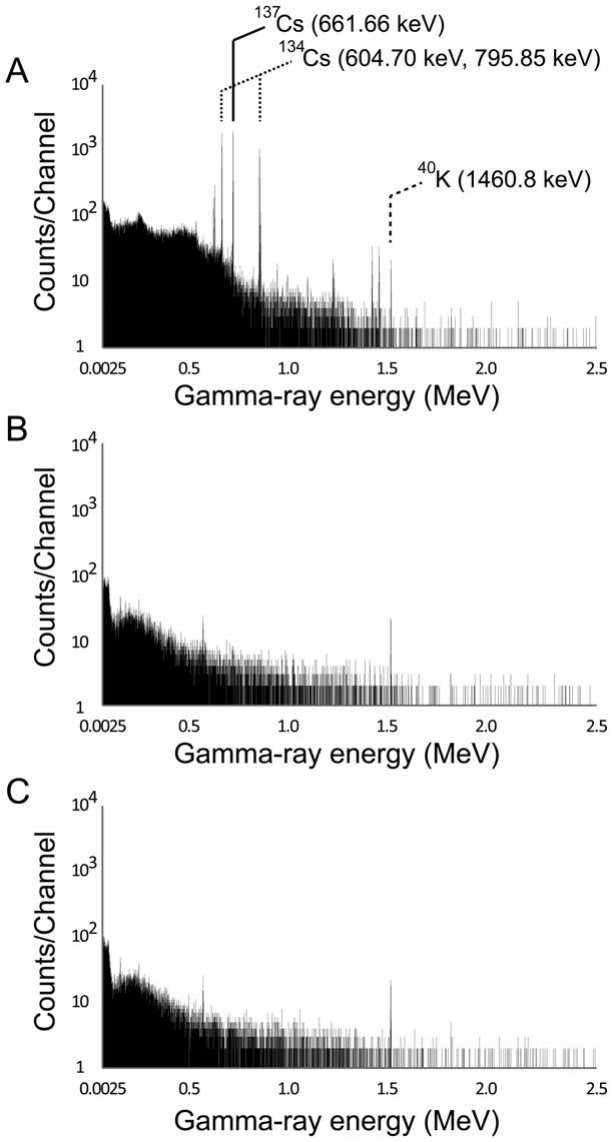
Typical gamma-ray spectra. (A, B) The spectra for the mature bamboo leaves sampled in Minamisouma (A) and Toyohashi (B). (C) The spectrum for an empty sample, showing the background. The ^40^K peak is estimated to be derived from concrete of buildings.

**Figure 3:**
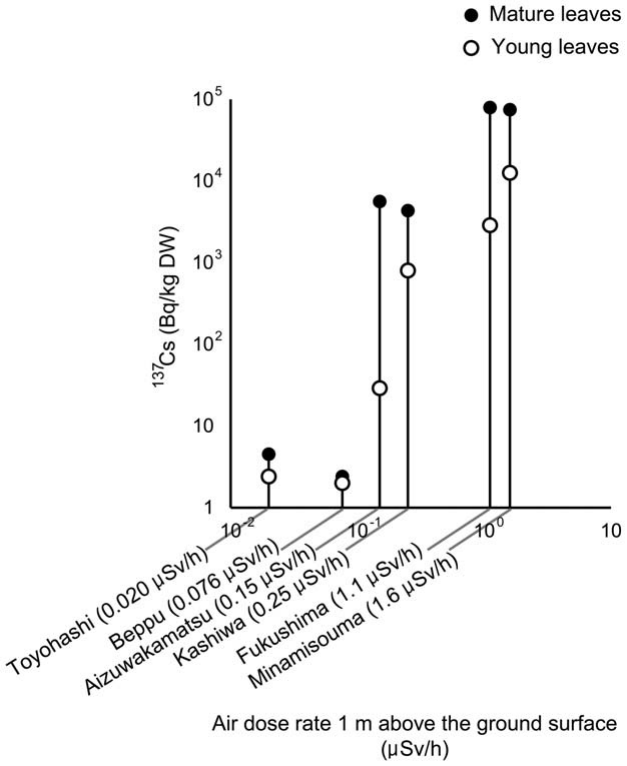
Relationship of ^137^Cs activities and the air dose rate 1 m above the ground surface in the sampling sites. The ^137^Cs activities in the samples from Toyohashi and Beppu were plotted at their measurable limits because they were below the limits.

Typical gamma-ray spectra from mature leaves are shown in Figure 2A and B. The ^134^Cs and ^137^Cs were clearly detected as peaks of gamma-ray energy in the samples obtained on 24^th^ July from Minamisouma city (Fig. 2A), but were below the measurable limits in the 20^th^ August samples from Toyohashi city (Fig. 2B). Unfortunately, we could not determine the quantity of ^40^K because of its low signal to noise ratio (Fig. 2), as ^40^K was not derived from the nuclear accident but from its natural occurrence at 0.0117 percent, and has an *in vivo* behavior similar to that of stable potassium. Table 1 shows the activity concentrations of bamboo leaves and branches from the various sampling sites from around early July to early September, 2011. Strikingly high activity concentrations were obtained in samples from Minamisouma and Fukushima cities near the Fukushima Daiichi nuclear plants. The highest ^134^Cs and ^137^Cs activities were 71.1 and 79.1 kBq/kg dry weight (DW) in mature leaves sampled in Fukushima city, respectively. In Aizuwakamatsu and Kashiwa cities, radiocesium activities were one order lower than those in Minamisouma and Fukushima cities. In Toyohashi and Beppu cities, radiocesium activities were below the measurable limits of up to 4.50 Bq/kg DW. The radiocesium activities tended to increase with the rise in air dose rates at the sampling sites (Fig. 3). In the radiocesium contaminated samples, leaves showed higher activities than branches (Table 1) and culms (Table 2) and, among these, mature and fallen leaves showed higher activities than young leaves (Table 1).

**Table 1:** The radioactivity concentrations of bamboo leaves and branches.

Material	Sampling date	Sampling area	Cs-134 (Bq/kg DW)	Cs-137 (Bq/kg DW)	Cs-134/Cs-137
Mature leaves of Hachiku	July 24.2011	Minamisouma city	63500±1790	74600±1890	0.851
Young leaves of Hachiku	July 24.2011	Minamisouma city	11100±969	12600±973	0.881
Branches of Hachiku	July 24.2011	Minamisouma city	10600±365	12300±367	0.862
Mature leaves of Azumanezasa	July 24.2011	Fukushima city	71100±1560	79100±1570	0.899
Young leaves of Azumanezasa	July 24.2011	Fukushima city	3080±747	2880±723	1.07
Branches of Azumanezasa	July 24.2011	Fukushima city	9130±338	10200±336	0.898
Mature leaves of Hoteichiku	July 24.2011	Aizuwakamatsu city	4690±610	5600±633	0.838
Young leaves of Hoteichiku	July 24.2011	Aizuwakamatsu city	<32.0	<29.0	-
Branches of Hoteichiku	July 24.2011	Aizuwakamatsu city	501±90.7	539±85.2	0.929
Mature leaves of Medake -1	July 1.2011	Kashiwa city	3440±224	4330±268	0.794
Young leaves of Medake -1	July 1.2011	Kashiwa city	764±109	801±115	0.953
Branches of Medake -1	July 1.2011	Kashiwa city	1140±65.0	1310±70.5	0.874
Mature leaves of Medake -2	July 21.2011	Kashiwa city	3230±242	3550±253	0.908
Young leaves of Medake -2	July 21.2011	Kashiwa city	857±124	922±118	0.930
Branches of Medake -2	July 21.2011	Kashiwa city	977±72.8	1150±76.1	0.852
Fallen leaves of Medake -1	July 21.2011	Kashiwa city	2790±202	3300±219	0.845
Fallen leaves of Medake -2	July 21.2011	Kashiwa city	2810±199	3240±216	0.867
Fallen leaves of Medake -3	July 21.2011	Kashiwa city	3200±235	3790±255	0.844
Mature leaves of Medake -1	August 20.2011	Toyohashi city	<4.50	<4.50	-
Young leaves of Medake -1	August 20.2011	Toyohashi city	<2.40	<2.40	-
Branches of Medake -1	August 20.2011	Toyohashi city	<0.600	<0.600	-
Mature leaves of Medake -2	August 20.2011	Toyohashi city	<3.80	<3.80	-
Young leaves of Medake -2	August 20.2011	Toyohashi city	<5.00	<5.00	-
Branches of Medake -2	August 20.2011	Toyohashi city	<1.50	<1.50	-
Mature leaves of Menyadake -1	September 5.2011	Beppu city	<2.40	<2.40	-
Young leaves of Menyadake -1	September 5.2011	Beppu city	<2.00	<2.00	-
Branches of Menyadake -1	September 5.2011	Beppu city	<0.400	<0.400	-
Mature leaves of Menyadake -2	September 5.2011	Beppu city	<2.80	<2.80	-
Young leaves of Menyadake -2	September 5.2011	Beppu city	<1.80	<1.80	-
Branches of Menyadake -2	September 5.2011	Beppu city	<0.400	<0.400	-

Because of space limitation, the species names are shown as Japanese names. Please refer to **Methods** for their scientific names.

**Table 2:** The radioactivity concentrations in the inner and outer layers of the culms of *Pleioblastus simonii* Nakai (Medake) sampled in Kashiwa city.

Material	Sampling date	Cs-134 (Bq/kg DW)	Cs-137 (Bq/kg DW)	Cs-134/Cs-137
Outer layer -1	August 24.2011	206±143	225±109	0.917
Outer layer -2	August 24.2011	96.8±38.0	136±59.9	0.711
Outer layer -3	August 24.2011	55.4±6.54	96.4±7.97	0.575
Inner layer -1	August 24.2011	249±67.7	282±74.7	0.885
Inner layer -2	August 24.2011	242±101	286±102	0.845
Inner layer -3	August 24.2011	107±27.2	118±26.0	0.903

To check whether the bamboos absorbed the radiocesium or not, we split the outer layer of the culms with a cutter knife to separate them into inner and outer culm layers. The activities within the inner layers were comparable to those in the outer layers (Table 2), suggesting radiocesium uptake.

## Discussion

In this study, we report the radiocesium contamination of bamboos sampled within a 25 to 980 km radius of the Fukushima Daiichi nuclear power plant. It has recently been reported that the ^134^Cs and ^137^Cs activity concentrations of azalea leaves collected in Chiba city, 220 km away from the Fukushima Daiichi, were 3.35 and 3.78 kBq/kg fresh weight, respectively [15]. These levels are comparable to our bamboo samples taken from Kashiwa city, 195 km away from the Fukushima Daiichi. Our results indicated that radiocesium contamination in mature and fallen leaves were higher than young leaves. It is possible that young leaves had not yet emerged at the time of the accident so that direct deposition from radiocesium fallout would have been negligible. On the other hand, the activity within the inner culm layers suggests the uptake of radiocesium by bamboo in Kashiwa, 195 km away from the Fukushima Daiichi (Table 2). Cesium is an alkaline metal, and is present as a monovalent cation in all soils. Because of its similarity with its nutrient analogue, potassium, a considerable amount of radiocesium is thought to have been taken up by the bamboos from contaminated soil. A recent study reported the uptake of radiocesium by emerged leaves of 14 plant species collected 220 km away from the Fukushima Daiichi, and suggested that the translocation velocity of radiocesium varied among the species tested [15]. Our results may be in accord with results from experimental field-grown rice plants that showed a lower translocation velocity of ^133^Cs than potassium [16].

The mature leaves of bamboo are often used to prepare bamboo grass tea. The radiocesium activity concentrations of the bamboo leaves from Minamisouma, Fukushima, Aizuwakamatsu and Kashiwa were over 500 Bq/kg, a temporary regulatory value decided by the Japanese Ministry of Health, Labour and Welfare (MHLW). As the effective dose coefficients for ingestion of ^134^Cs and ^137^Cs are 0.019 and 0.013 µSv/Bq, the effective doses from the 200 mg amounts that are roughly used for a cup of tea prepared from milled tea powder from Fukushima and Kashiwa cities would be 0.476 and 0.0243 µSv, respectively. If just six cups of the bamboo grass tea from Fukushima city are consumed every day, the radiation dose from the tea would be in excess of 1 mSv/year. Therefore, we consider it preferable to avoid the regular drinking of bamboo tea from highly contaminated areas surrounding the Fukushima nuclear plant.
